# Factors Affecting Cancer Mortality in Young Adults: Findings from a Prospective Cohort Study

**DOI:** 10.3390/cancers16223853

**Published:** 2024-11-17

**Authors:** Ngoan T. Le, Yen T.-H. Pham, Linh T. Le, Hang V. Dao, Chihaya Koriyama, Toan H. Ha, Maureen Lichtveld, Suresh V. Kuchipudi, Nhi Y.-N. Huynh, Dai D. Nguyen, Hung N. Luu

**Affiliations:** 1 Institute of Research and Development, Duy Tan University, Da Nang 550000, Vietnam; 2 Department of Occupational Health, Institute for Preventive Medicine and Public Health, Hanoi Medical University, Hanoi 100000, Vietnam; 3 University of Pittsburgh Medical Center, Hillman Cancer Center, Pittsburgh, PA 15261, USA; phamy@upmc.edu; 4 Department of Epidemiology, School of Public Health, University of Pittsburgh, Pittsburgh, PA 15261, USA; 5 Laboratory of Embryology and Genetics of Human Malformation, Imagine Institute, INSERM UMR, 59045 Paris, France; lelinh2611@gmail.com; 6 Internal Medicine Faculty, Hanoi Medical University, Hanoi 100000, Vietnam; hangdao.fsh@gmail.com; 7 Department of Epidemiology and Preventive Medicine, Graduate School of Medicine and Dental Science, Kagoshima University, Kagoshima 890-0065, Japan; fiy@m.kufm.kagoshima-u.ac.jp; 8 Department of Infectious Disease and Microbiology, School of Public Health, University of Pittsburgh, Pittsburgh, PA 15261, USA; toan.ha@pitt.edu (T.H.H.); skuchipudi@pitt.edu (S.V.K.); 9 Department of Environmental and Occupational Health, School of Public Health, University of Pittsburgh, Pittsburgh, PA 15261, USA; mlichtve@pitt.edu; 10 School of Medicine, International University of Health and Welfare, Narita 324-8501, Japan; 17a1101@g.iuhw.ac.jp (N.Y.-N.H.); 17a1037@g.iuhw.ac.jp (D.D.N.)

**Keywords:** risk factors, cancer mortality, prospective cohort study, young adults, Vietnam

## Abstract

Background/Objectives: Cancer incidence in young adults or those aged 15–49 years old has increased during the past decade. Knowledge about the risk factors for cancer-related deaths in young adults is limited, particularly in low- and middle-income countries (LMICs). Methods: This analysis was based on the Hanoi Prospective Cohort Study, an ongoing study of 39,401 participants aged 15 or older in Northern Vietnam in the 2007–2019 period. A Cox proportional hazard regression model was used to calculate the hazard ratio (HR) and 95% confidence intervals (95% CIs) for the association between potential factors and the risk of cancer-related deaths. Results: With a median follow-up of 11.01 years, we identified 164 deaths in young adults out of 554 total deaths. Overall, family history of cancer (HR = 7.34; 95% CI: 3.30–16.36), drinking alcohol (HR = 1.82; 95% CI: 1.18–2.81), and smoking (HR = 2.22; 95% CI: 1.36–3.63) were found to be risk factors, while drinking coffee was found to be a protective factor (HR = 0.49; 95% CI: 0.24–1.00) for cancer-related deaths in young adults. Young male adults were found to be at a higher risk due to excessive cigarette smoking (HR = 1.91; 95% CI: 1.00–3.68) and alcohol consumption (HR = 2.15; 95% CI: 1.32–3.53) than those aged 50 years and older (HR = 1.36 and 95% CI: 0.96–1.93 and 1.27 and 95% CI: 0.97–1.67, respectively). The risk of death from cancer in women compared with men in the young population was twice as high as that in the older population (HR = 1.18 and 95% CI: 0.72–1.94 vs. 0.47 and 95% CI: 0.35–0.63, respectively). Conclusions: Our data suggest that the young Vietnamese population is vulnerable to the risk of cancer-related deaths and that cancer in women will increase rapidly in the future.

## Simple Summary

The incidence of cancer in young adults has been increasing worldwide, yet information on its risk factors in low- and middle-income countries (LMICs) is limited. This analysis of more than 39,000 participants in Vietnam, an LMIC in Southeast Asia, shows that the risk of cancer-related deaths caused by excessive smoking, particularly waterpipe smoking, and drinking alcohol in young adults (or individuals less than 50 years of age) is much higher than the risk for individuals aged 50 years or older, and the risk of death for women is almost two-folds higher than that for men younger than 50, whereas the risk of cancer-related death in women aged ≥50 is significantly lower than that in men. This suggests that the young Vietnamese population is vulnerable to the risk of cancer-related deaths.

## 1. Introduction

Cancer among young adults, or early-onset cancer, is defined as cancer occurring among adults aged between 18 and 49 years [1]. Worldwide, there were 1.8 million cancer cases and close to 400,000 deaths due to cancer among young adults [2]. Even though cancer is a disease among older individuals or those who are 50 years of age or older, cumulative evidence shows that there has been an increased trend in cancer incidence among young adults [1,2]. Recent efforts have shown increased incidence trends in colorectal cancer (CRC) [3,4,5], pancreatic cancer [6], and gastric cancer [7] in young adults in the U.S. and worldwide. The reasons for this rising trend in cancer incidence among young adults are unclear. Early data suggest that young adults might be at a greater risk due to their high level of susceptibility to human-made or natural environmental exposures as their bodies are not grown enough to protect them against carcinogens [8,9,10]. However, several factors are thought to have contributed to this phenomenon, such as obesity, smoking, diabetes, physical inactivity, microbiota, sleep patterns, or transient exposure to carcinogenic compounds [11,12,13,14,15,16]. Identifying such factors for cancer incidence and mortality in young adults plays a crucial role in the healthcare system given the long-term impacts, such as infertility, cardiovascular diseases, and secondary cancers [8,9,10].

Vietnam is a low- and middle-income country (LMIC) with a total population of close to 100 million, and it is one of the most populous countries worldwide. During the past two decades, the Vietnamese economy has grown sharply [17], resulting in improved life expectancy and a widespread adoption of Western lifestyles. Consequently, there have been swift increases in cancer incidence and mortality in Vietnam [18]. Recent data show that in 2020, there were 122,690 cancer-related deaths in Vietnam, making up 20% of the total deaths in the country [19,20]. Vietnam also has a different age structure compared with Western countries and others, with 22.6% of individuals being in the age range of 0–14 years, 15.2% being in the age range of 1–24 years, and 45.7% being in the age range of 25–54 years [21]. Studies have been conducted in Western countries to identify the risk factors for cancer in young adults. For instance, several large prospective cohort studies in the U.S. (i.e., the Nurses’ Health Study or Health Professionals Follow-up Study) identified metabolic dysregulation [22] and a sulfur microbial diet [23] (food intake associated with the enrichment in sulfur-metabolizing microbes in humans) as risk factors and vitamin D intake [24] as a protective factor for early-onset colorectal cancer. Other risk factors were also identified, including cancer history in a first-degree relative, hyperlipidemia, obesity, alcohol consumption, smoking, a sedentary lifestyle, consumption of processed meat, and Western dietary patterns [25,26]. Nonetheless, data from LMICs, including Vietnam, are limited. Because there is a difference in lifestyle between Western and Eastern countries, it is postulated that the risk factors for cancer incidence and/or mortality in young adults in LMICs might be different from those in Western countries.

In this analysis, we determined the risk factors for cancer-related mortality in Vietnam using data from the Hanoi Prospective Cohort Study, the first-ever and ongoing prospective cohort study among more than 52,000 participants in Vietnam.

## 2. Materials and Methods

### 2.1. Study Population

The current analysis used data from the Hanoi Prospective Cohort Study (HPCS), which were previously described in detail [27]. Briefly, the HPCS is an ongoing population-based prospective cohort study, which recruited 52,325 Vietnamese participants who were one year old or older between April 2007 and November 2008. Recruited participants were residents living in five urban communities in Hanoi, the capital city; three in rural communities in Hung Yen province; and one in a mountainous region in Phu Tho province, all of which are located in Northern Vietnam. Children under 15 years of age were included in the HPCS because injury mortality accounted for approximately 12% of the total deaths in Vietnam during that period [28], and smoking increased the injury risk [29]. The HPCS was approved by the Institutional Review Boards of the Hanoi Medical University and the International University of Health and Welfare, Japan.

At baseline, study participants were interviewed by trained interviewers using structured questionnaires to obtain information on sociodemographics, body weight and height, lifetime use of tobacco, medical history, family history of cancer, and diet. In the current analysis, we excluded 7.56% of the total eligible participants, including 9944 participants who were under 15 years old and 2980 participants who migrated out of the areas in the first year of follow-up. Because we could not report the date of migration, these migrated participants were excluded. The final sample for our current analysis included 39,401 study participants, including 27,705 participants aged 15–49 years (or young adults), of whom 164 died due to cancer and 27,541 survived, and 11,696 participants aged 50 or older, of whom 390 died due to cancer and 11,306 survived (Supplementary Figure S1).

### 2.2. Dietary Assessment

Dietary information was collected using the semi-quantitative food frequency questionnaire (FFQ), which was prepared by the Vietnam National Institute of Nutrition. This FFQ was used to obtain the average frequency of food intake and portion sizes from all study participants during the past 12 months for 85 food items and food groups commonly consumed by Vietnamese.

The semi-quantitative FFQ was first designed in 2003 based on the results of a 2000 National Dietary Survey asking for records of food consumption in the past 24 h for three consecutive days in 158 households (or 741 individuals). Food selection for the FFQ from the 2000 National Dietary Survey results contributed up to 90% or higher essential nutrients converted from the natural organic whole food used by the participating households [30,31,32].

Study participants were asked how frequently they consumed a food and food group in 6 categories, ranging from *“6–11 times/year”* to *“1–3 times/month”, “1–2 times/week”, “3–4 times/week”, “5–6 times/week”*, and *“1–3 times/day”*, followed by a question on the amount of food consumed from three portion sizes (i.e., small or 75% of mean intake, mean intake, and large or 125% of mean intake). The average daily intake of 95 nutrients and non-nutrient compounds was calculated for each participant using the Vietnamese Food Composition Database [33]. The FFQ was validated against two 24 h dietary recalls (24-HDRs), one on a weekday and one on three consecutive other days, among 298 families (or 1327 individuals). The Pearson correlation coefficients between the FFQ and 24-HDR for the majority of calorie-adjusted nutrients ranged from 0.20 (for lipids) to 0.53 (for energy intake) [30].

### 2.3. Smoking Assessment

Two main types of tobacco, including cigarette and waterpipe, were collected in HPCS [34]. Detailed information regarding the smoking assessment is described in detail in the Supplementary Methods.

### 2.4. Cancer-Related Deaths Ascertainment

Mortality information (i.e., cancer-related mortality) was identified using medical records. We used the International Classification of Diseases Tenth Revision (ICD-10) codes for cause-specific mortality. The completeness, sensitivity, and specificity were 93.9%, 75.4%, and 98.4% for the initial mortality registry by the state commune health station (CHS). The list and CHS’s underlying cause of death were cross-checked with mortality reports by the Office of Population Family Planning and the Justice office’s records to avoid under- and double-mortality registration (details in the Supplementary Methods) [35].

### 2.5. Assessment of Other Potential Risk Factors

Several potential confounding or risks for death were identified in prior studies [27,36,37]. The following potential risk factors were included in the current analysis: (1) sex (i.e., male vs. female), (2) education level (i.e., primary and secondary or higher), (3) body mass index (BMI) (i.e., <18.5, 18.5 to <23, ≥23 kg/m^2^), (4) alcohol consumption (i.e., yes vs. no), (5) family history of cancer (i.e., yes vs. no), (6) smoking status (i.e., never vs. ever), (7) types of tobacco smoking (i.e., never smoker, dual smoker of cigarette and waterpipe, exclusive waterpipe, exclusive cigarette), (8) coffee drinking status (i.e., ever vs. never), (9) history of diabetes (i.e., yes vs. no), (10) total energy intake (Kcal/day), (11) protein intake (g/day), (12) fat intake (g/day), (13) carbohydrate intake (g/day), (14) fiber intake (g/day), (15) meat intake (g/week), (16) poultry intake (g/week), (17) bean intake (g/week), (18) vegetables intake (g/week), (19) fruits intake (g/week), (20) eggs intake (g/week), and (21) fish intake (g/week).

### 2.6. Statistical Analysis

Mean and standard deviation (SD) were used to analyze continuous variables, while counts and proportions were used for the analysis of categorical variables. We also used the *t*-test and *χ^2^* test to compare the difference in distributions of continuous and categorical variables, respectively, between cancer-related deaths and survived participants. Person-years at risk for each participant were calculated from the date of the baseline interview to the date of death, migration out of communities, or 31 December 2019 (i.e., the last day of follow-up), whichever occurred first.

The Cox proportional hazard regression method was used to determine potential risk factors for cancer-related deaths among young adults (15–49 years of age), those who were 50 years of age and older, and the entire cohort (both 15–49 years of age and ≥50 years of age) with the aforementioned variables (or factors) included in the models. We calculated hazard ratios (HRs) and corresponding 95% confidence intervals (CIs) for the risk of death according to such factors. To further understand the population attributable risk fraction (in percentage) (PAF) in young adults and those ≥50 years of age, for each group, we used the following formula, where *p_e_* represents the prevalence of exposure in young adults and/or people 50 or older under this study and HR is the adjusted hazard ratio [38].

$$PAF=\frac{pe\times \left(HR-1\right)\times 100}{\left(pe\times (HR-1\right)+1)}$$

All statistical analyses were performed using the Stata package, version 14.0 (StataCorp LP., College Station, TX, USA). All *p-*values presented are two-sided, and *p-*values < 0.05 were considered statistically significant.

## 3. Results

**Figure 1:**
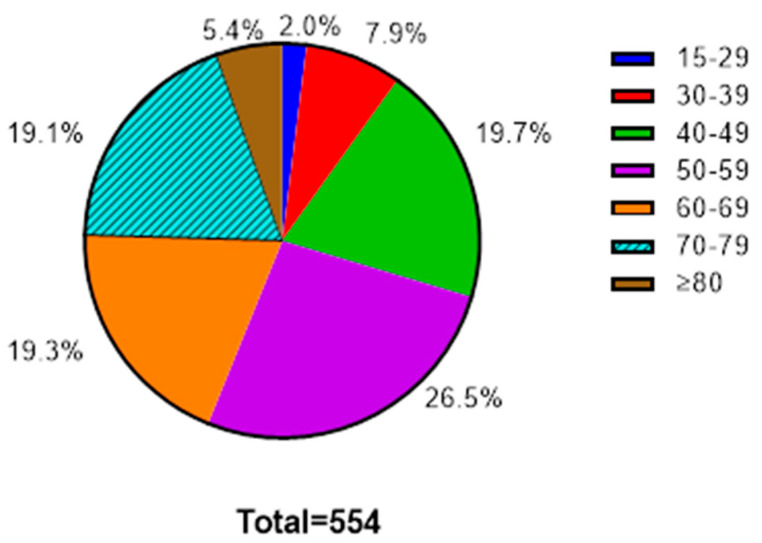
Distribution of cancer-related deaths by age group in the Hanoi Prospective Cohort Study.

We identified 164 cancer-related deaths among 27,705 young participants and 390 cancer-related deaths among 11,696 individuals aged 50 years and older. Among the 554 cancer-related deaths found in our cohort, 2% were among individuals aged 15–29, 7.9% were among those aged 30–39, and 19.7% were among those aged 40–49 (Figure 1).

Among young adults, compared to survived participants, cancer-related deceased individuals were more likely to have a family history of cancer and more likely to be ever-alcohol drinkers (all *p*’s < 0.05). There was no significant difference between cancer-related deaths and survival participants in distributions of education level, refrigerator use, BMI, coffee drinking status, history of diabetes, energy intake, or carbohydrate intake. Similar observations were observed in participants aged 50 years and older (Table 1).

Overall, family history of cancer, alcohol drinking status, tobacco smoking status, and carbohydrate intake were risk factors. In contrast, coffee drinking status was a protective factor for cancer-related death among young adults. The respective HRs and 95% CIs were 7.34 (3.30–16.36), 1.82 (1.18–2.81), 2.22 (1.36–3.63), 1.39 (1.07–1.81), and 0.49 (0.24–1.00). Also, dual smokers of cigarettes and waterpipes (HR = 2.12, 95% CI: 1.17–3.83), exclusive cigarette smokers (HR = 2.94, 95% CI: 1.71–6.08), and exclusive waterpipe smokers (HR = 2.94, 95% CI: 1.71–5.08) were risk factors for cancer-related deaths among young adults. The association between types of tobacco smoking and cancer-related death was also more robust in men (dual smoker: HR = 2.42, 95% CI: 1.26–4.64; exclusive waterpipe smoker: HR = 3.51, 95% CI: 1.90–6.47; exclusive cigarette smoker: HR = 1.91, 95% CI: 1.00–3.68) (Table 2).

Among participants aged 50 years and older, being a woman was a protective factor for cancer-related death (HR = 0.47, 95% CI: 0.35–0.63). Also, family history of cancer (HR = 3.97, 95% CI: 2.15–7.32), ever smoker (HR = 1.48, 95% CI: 1.13–1.93), exclusive waterpipe smoker (HR = 1.66, 95% CI: 1.22–2.28), and having a history of diabetes (HR = 2.62, 95% CI: 1.68–4.33) were risk factors for cancer-related death in this population (Table 3). Young male adults appeared to be at higher risk due to dual smoking (HR = 2.42, 95% CI: 1.26–4.64), exclusive cigarette smoking (HR = 1.91, 95% CI: 1.00–3.68), and alcohol drinking (HR = 2.15, 95% CI: 1.32–3.53) than those aged 50 years and older ((HR = 1.38, 95% CI: 0.94–2.04), (HR = 1.36, 95% CI: 0.96–1.93), and (HR = 1.27, 95% CI: 0.97–1.67), respectively) (Table 2 and Table 3). In the analysis of our entire cohort, being a woman and being a coffee drinker were protective factors. In contrast, a family history of cancer, being an alcohol drinker, being a tobacco smoker (any type), and having a history of diabetes were risk factors for cancer-related deaths (Table 4).

The population attributable fraction in young adults was higher than the people 50 or higher by 25.18% (39.25% versus 14.06%) and 14.52% (38.97% versus 24.45%) for alcohol usage and ever tobacco smoking, respectively (Supplementary Table S1). The proportion of survival was lower in alcohol drinkers than no alcohol drinkers in both young adults aged under 50 (A) and those aged 50 years and older (B) and exclusive waterpipe smokers in young (C) and older (D), respectively (Supplementary Figure S2).

## 4. Discussion

In a first-ever, large, and ongoing prospective cohort study of more than 52,000 Vietnamese, we found that family history of cancer, ever alcohol drinking status, ever tobacco smoking status, and high intake of carbohydrates were risk factors while ever-coffee drinking status was a protective factor for cancer-related death among young adults (or those who were aged 15–49). We also found that among participants aged 50 years or older, a family history of cancer, an ever smoker, and having a history of diabetes were risk factors. The risk of death from cancer in women was almost as high as that in men in the younger group of 15–49 years of age, while in the group of women ≥50, it was significantly lower than in men.

While there have been extensive studies on the risk factors for cancer incidence in young adults, studies on the risk factors for cancer-related death are limited. Consequently, finding a comparable study to ours is a challenge, and we could only find those studies of cancer incidence that resonated with our findings in cancer mortality. We found that a family history of cancer was a risk factor for cancer-related death in our cohort, which might be in line with findings from a cohort study of more than 376,000 relatives of early-onset cancer patients diagnosed between 1970 and 2012, in which they found strong evidence that a family history of cancer was associated with early-onset of colorectal cancer [39]. Another study using the Utah Pedigree Database recently confirmed that the risk of early-onset colorectal cancer (CRC) was elevated among first- and second-degree relatives of early-onset CRC cases [40]. In a case-control study of 269 early-onset CRC cases vs. 1122 controls in New York City, the authors found that a person with a family history of CRC had close to 9 times higher odds of having early-onset CRC than a person without a family history of CRC (OR = 8.61, 95% CI: 4.83–15.75) [41]. Finally, a recent meta-analysis of 20 studies found first-degree relatives were a risk factor for CRC risk [25].

We also found that ever smoking status was the leading cause of cancer-related deaths in young adults in our cohort, followed by ever alcohol drinking status. Again, while there are no comparable data to ours, findings from prior studies on cancer incidence can be used as references. Accordingly, in a population-based cohort study using a national database of electronic medical records (EHRs), called Explorys, Syed et al. [42] found both tobacco use (OR = 2.46, 95% CI: 2.33–2.59) and alcohol use (OR = 1.71, 95% CI: 1.62–1.80) to be risk factors for early-onset colorectal cancer. A recent meta-analysis of 20 studies [25], including 10 case-control studies, eight cohort studies, and two cross-sectional studies, found that alcohol drinking was a risk factor, while smoking was a suggestive risk factor for CRC risk. The respective relative risks (RRs) and 95% CIs were 1.71 (1.62–1.80) and 1.35 (0.81–2.25).

A unique and interesting finding from our cohort is that waterpipe smoking, either in a dual type of waterpipe and/or cigarette or exclusive waterpipe, was a strong risk factor for cancer-related death in young adults in the Vietnamese population with the highest risk among exclusive waterpipe smokers, compared with never smokers. The prevalence of waterpipe smoking in Vietnam is 6.4%, relatively higher than that in other countries in the region (i.e., 3.3%) [43]. Prior biomarker studies have shown that waterpipe smokers receive much higher amounts of PAH, benzenes, and nicotine than cigarette smokers, all carcinogenic agents [43,44,45]. Dietary carbohydrate intake was also found to be a risk factor for cancer-related death among young adults in our cohort, which appears contrary to prior studies. For instance, in a recent meta-analysis of 17 studies, including eight case-control studies and nine cohort studies, Huang et al. [46] found that dietary intake of carbohydrates was not associated with the risk of colorectal cancer (RR = 1.08, 95% CI: 0.93–1.23); however, in a stratified analysis, carbohydrates were actually a risk factor for CRC incidence among men (RR = 1.23, 95% CI: 1.01–1.57). A high carbohydrate intake was associated with increased risk of mortality in the Atherosclerosis Risk in Communities (ARIC) study and several other cohort studies in the meta-analysis, including the Nurses’ Health Study and Health Professionals Follow-up Study as well as the European Prospective Investigation into Cancer and Nutrition Study (EPIC) [47].

We did not find BMI to be a risk factor for cancer-related mortality in our cohort, which might be inconsistent with prior findings in cancer incidence studies. For instance, Liu et al. [11] used data from the Nurses’ Health Study (NHS) to report that weight gain in early childhood among women was significantly associated with an increased risk of early-onset colorectal cancer. Compared to women with a normal BMI, individuals with a BMI of 30 or higher had the highest risk of early-onset colorectal cancer (RR = 1.93, 95% CI: 1.15–3.25). The recent meta-analysis of 20 studies by O’Sullivan et al. [25] also found BMI to be a risk factor for early-onset colorectal cancer (RR = 1.54, 95% CI: 1.01–2.35), which is also inconsistent with our findings. Different population structures and settings might be one of the reasons for this inconsistency. Further research is thus needed to confirm our findings.

We did not find a history of diabetes to be a risk factor for cancer-related death in the young Vietnamese adults in our analysis, a finding that is inconsistent with a recent meta-analysis of 19 studies (i.e., 12 case-control studies and seven cohort studies) that found a significant association between type 2 diabetes and the risk of early-onset CRC (OR = 1.43, 95% CI: 1.08–1.80) [48]. However, in a large case-control study, involving 1953 CRC cases and 4154 controls, La Vecchia et al. [49] observed that in those who had diabetes for less than 10 years, there was a null association between diabetes and CRC. As diabetes is an age-related disease, diabetes might not be a strong risk factor for cancer-related mortality among young adults, which is similar to findings from a recent case-control study of 651 early-onset CRC cases and 67,416 controls using the U.S. Veterans Health Administration database [50]. Further study is therefore warranted to confirm our findings in other populations and settings.

Finally, there was no sex difference in terms of the risk of cancer-related deaths among the young people in our cohort. This finding is also in contrast to the recent meta-analysis by O’Sullivan et al. [25], in which they reported that being male had a 1.6-fold increased risk of early-onset CRC compared with being female. On the contrary, we also showed that being a woman was a protective factor among participants aged 50 years or older. Further works are thus warranted to replicate our findings in different prospective cohort studies.

Our study has several strengths, including a prospective design, a large sample size, and detailed information about lifestyle, including dietary factors and alcohol and tobacco smoking habits [27]. Such detailed information allowed us to use them as covariates in the multivariable models. Our study also has several limitations, including the misclassification of smoking status and dietary pattern because study participants self-reported and/or recalled answering questions. Also, even though the findings of the waterpipe and related information are intriguing, we would not be able to evaluate its impact over time because such information was only collected at enrollment (or baseline).

## 5. Conclusions

In summary, in a first-ever prospective cohort study of Vietnamese participants, we found that family history of cancer, alcohol drinking status, tobacco smoking status, and carbohydrate intake were risk factors and coffee drinking status was a protective factor for cancer-related death among young adults. We observed that the young generation aged under 50 is at greater risk of cancer mortality due to alcohol drinking and waterpipe and cigarette smoking than those aged 50 years and older. Further studies, particularly in LMICs and similar settings, are thus warranted to confirm our findings.

## Figures and Tables

**Table 1:** Selected baseline characteristics of study participants in the Hanoi Prospective Cohort Study.

	Young Adults (15–49 Years Old)			Adults ≥ 50 Years Old		
	Cancer-Related Deaths (*n* = 164) (*n*, %)	Survived Participants (*n* = 27,541) (*n*, %)	*p*-Value	Cancer-Related Deaths (*n* = 390) (*n*, %)	Survived Participants (*n* = 11,306) (*n*, %)	*p*-Value
Sex						
Male	108 (65.8)	13,478 (48.9)	<0.0001	269 (69.0)	4930 (43.6)	<0.0001
Female	56 (34.2)	14,063 (51.1)		121 (31.0)	6376 (56.4)	
Highest level of education ^a^						
Primary school	16 (9.8)	1992 (7.2)	0.21	167 (42.8)	5498 (48.6)	0.11
Secondary school or higher	148 (90.2)	25,549 (92.8)		223 (57.2)	5808 (51.4)	
Refrigerator use ^a^						
No	80 (48.8)	13,659 (49.6)	0.83	141 (36.1)	4545 (40.2)	0.02
Yes	84 (51.2)	13,882 (50.4)		249 (63.9)	6761 (59.8)	
Family history of cancer ^a^						
No	157 (95.7)	27,083 (98.3)	0.01	379 (97.2)	11,195 (99.0)	<0.0001
Yes	7 (4.3)	458 (1.7)		11 (2.8)	111 (1.0)	
BMI, (Mean ± SD) ^a^	20.0 ± 2.2	19.9 ± 8.4	0.41	19.8 ± 2.6	20.9 ± 4.6	0.13
<18.5	52 (31.7)	10,695 (38.8)	0.17	157 (40.3)	4684 (41.4)	0.26
18.5–22.9	99 (60.4)	14,965 (54.3)		200 (51.3)	5419 (47.9)	
≥23	13 (7.9)	1881 (6.9)		33 (9.4)	1203 (10.7)	
Alcohol drinking status ^a^						
Never	80 (48.8)	22,005 (79.9)	<0.0001	199 (51.0)	7939 (70.2)	<0.0001
Ever	84 (51.2)	5536 (20.1)		191 (46.0)	3367 (29.8)	
Coffee drinking status ^a^						
Never	155 (94.5)	25,250 (91.7)	0.19	362 (92.8)	10,427 (92.2)	0.67
Ever	9 (5.5)	2291 (8.3)		28 (7.2)	879 (7.8)	
History of diabetes						
No	163 (99.4)	27,503 (99.9)	0.11	374 (95.9)	11,130 (98.4)	<0.0001
Yes	1 (0.6)	38 (0.1)		16 (4.1)	176 (1.6)	
Energy intake (Kcal/day, Mean ± SD)	1767.5 ± 417.2	1771.7 ± 426.9	0.45	1775.3 ± 436.2	1733.4 ± 423.2	0.02
Protein intake (g/day, Mean ± SD)	63.4 ± 17.8	66.0 ± 18.5	0.04	63.8 ± 17.9	63.6 ± 18.8	0.43
Fat intake (g/day, Mean ± SD)	21.8 ± 8.9	24.2 ± 10.1	0.001	22.6 ± 9.8	22.8 ± 10.3	0.35
Carbohydrate intake (g/day, Mean ± SD)	333.0 ± 83.4	326.0 ± 86.3	0.15	332.8 ± 86.1	321.9 ± 83.9	0.006
Fiber intake (g/day, Mean ± SD)	3.3 ± 0.9	3.3 ± 0.9	0.03	3.3 ± 0.9	3.2 ± 0.9	0.03

^a^ Based on the reported data, standard deviation (SD), body mass index (BMI).

**Table 2:** Risk factors for cancer-related deaths in young adults (15–49 years of age), overall and stratified by sex, based on the Hanoi Prospective Cohort Study.

	Overall		Men		Women	
	# Deaths/ Person-Years	Multivariable Model HR (95% CI)	# Deaths/ Person-Years	Multivariable Model HR (95% CI)	# Deaths/ Person-Years	Multivariable Model HR (95% CI)
Sex						
Men	108/149,570	1.00	-	-	-	-
Women	56/156,469	1.18 (0.72, 1.94)	-	-	-	-
Highest level of education						
Primary school	16/21,830	1.00	11/11,441	1.00	5/10,389	1.00
Secondary school or higher	148/284,209	1.09 (0.64, 1.83)	97/138,129	1.09 (0.58, 2.05)	51/146,080	1.08 (0.43, 2.73)
Family history of cancer						
No	157/300,961	1.00	106/147,074	1.00	51/153,887	1.00
Yes	7/5078	**7.34 (3.3, 16.36)**	2/2496	3.87 (0.92, 16.25)	5/2582	**15.43 (5.86, 40.64)**
BMI						
18.5–22.9	99/166,547	1.00	65/81,265	1.00	34/85,282	1.00
<18.5	13/20,932	0.80 (0.45, 1.44)	11/13,451	0.83 (0.44, 1.58)	2/7480	0.52 (0.12, 2.17)
≥23	52/118,560	1.00 (0.71, 1.40)	32/54,854	0.94 (0.61, 1.44)	20/63,707	1.01 (0.58, 1.76)
Alcohol drinking status						
Never drinker	80/244,508	1.00	24/92,104	1.00	56/152,405	1.00
Ever drinker	84/61,531	**1.82 (1.18, 2.81)**	84/57,466	**2.15 (1.32, 3.53)**	0/4064	-
Coffee drinking status						
Never drinker	155/280,527	1.00	100/134,102	1.00	55/146,425	1.00
Ever drinker	9/25,512	**0.49 (0.24, 1.00)**	8/15,468	0.69 (0.33, 1.44)	1/10,044	**0.12 (0.01, 0.95)**
Tobacco smoking status						
Never	74/242,767	1.00	18/88,283	1.00	-	-
Ever	90/63,272	**2.22 (1.36, 3.63)**	90/61,287	**2.55 (1.46, 4.46)**	-	-
Type of tobacco smoking					-	-
Never smoker	74/242,767	1.00	18/88,283	1.00	-	-
Dual smoker	27/19,644	**2.12 (1.17, 3.83)**	27/19,112	**2.42 (1.26, 4.64)**	-	-
Exclusive waterpipe	40/18,934	**2.94 (1.71, 5.08)**	40/18,261	**3.51 (1.90, 6.47)**	-	-
Exclusive cigarette	23/24,694	**1.66 (0.91, 3.02)**	23/23,914	**1.91 (1.00, 3.68)**	-	-
History of diabetes						
No	163/305,630	1.00	107/149,350	1.00	56/156,281	1.00
Yes	1/1409	2.10 (0.29, 15.09)	1/220	3.88 (0.54, 27.97)	0/188	-
Energy (Kcal/day) *	164/336,509	0.76 (0.54, 1.07)	108/180,040	1.02 (0.90, 1.17)	56/156,469	**0.53 (0.30, 0.95)**
Protein (g/day) *	164/336,509	1.16 (0.88, 1.54)	108/180,040	1.00 (0.71, 1.40)	56/156,469	1.60 (0.98, 2.61)
Fat (g/day) *	164/336,509	0.77 (0.63, 0.93)	108/180,040	0.80 (0.63, 1.01)	56/156,469	**0.70 (0.50, 0.99)**
Carbohydrate (g/day) *	164/336,509	**1.39 (1.07, 1.81)**	108/180,040	0.98 (0.81, 1.20)	56/156,469	1.46 (0.93, 2.27)
Fiber (g/day) *	164/336,509	0.97 (0.82, 1.14)	108/180,040	0.97 (0.82, 1.14)	56/156,469	0.92 (0.70, 1.22)
Meat (g/week) *	164/336,509	0.90 (0.77, 1.05)	108/180,040	0.89 (0.73, 1.08)	56/156,469	0.92 (0.70, 1.22)
Poultry (g/week) *	164/336,509	0.95 (0.84, 1.07)	108/180,040	0.87 (0.75, 1.02)	56/156,469	1.12 (0.91, 1.38)
Beans (g/week) *	164/336,509	1.05 (0.93, 1.17)	108/180,040	1.03 (0.89, 1.18)	56/156,469	1.08 (0.89, 1.31)
Vegetables (g/week) *	164/336,509	1.06 (0.90, 1.24)	108/180,040	1.05 (0.87, 1.28)	56/156,469	1.07 (0.82, 1.40)
Fruits (g/week) *	164/336,509	1.02 (0.89, 1.17)	108/180,040	1.09 (0.92, 1.29)	56/156,469	0.89 (0.70, 1.13)
Eggs (g/week) *	164/336,509	1.11 (0.99, 1.25)	108/180,040	1.11 (0.96, 1.28)	56/156,469	1.13 (0.92, 1.38)
Fish (g/week) *	164/336,509	0.97 (0.84, 1.12)	108/180,040	1.00 (0.84, 1.19)	56/156,469	0.93 (0.73, 1.19)

Body mass index (BMI, Asian criteria-based BMI), hazard ratio (95% confidence interval) HR (95%CI). Multivariable models include indicators (if applicable) of sex, age groups, education, BMI, family history of cancer, alcohol drinking status, coffee drinking status, smoking status, history of diabetes, use of fridge at home, total energy intake, protein intake, fat intake, carbohydrate intake, and fiber intake. * Continuous scale of quintiles. **Bold** font: statistical significance (*p* < 0.05)

**Table 3:** Risk factors for cancer-related deaths in adults ≥50 years of age, overall and stratified by sex, based on the Hanoi Prospective Cohort Study.

	Overall		Men		Women	
	# Deaths/ Person-Years	Multivariable Model HR (95% CI)	# Deaths/ Person-Years	Multivariable Model HR (95% CI)	# Deaths/ Person-Years	Multivariable Model HR (95% CI)
Sex						
Men	269/52,875	1.00				
Women	121/67,911	**0.47 (0.35, 0.63)**				
Highest level of education						
Primary school	46,205	1.00	74/13,522	1.00	67/32,683	1.00
Secondary school or higher	74,580	0.97 (0.76, 1.23)	195/39,353	1.00 (0.74, 1.35)	54/35,227	0.95 (0.61, 1.48)
Family history of cancer						
No	379/119,560	1.00	260/52,448	1.00	119/67,111	1.00
Yes	11/1226	**3.97 (2.15, 7.32)**	9/426	**5.64 (2.87, 11.1)**	2/799	1.63 (0.38, 6.92)
BMI						
18.5–22.9	200/58,790	1.00	141/26,767	1.00	59/32,023	1.00
<18.5	33/13,152	0.70 (0.49, 1.02)	17/6927	0.48 (0.29, 0.80)	16/6224	1.48 (0.85, 2.58)
≥23	157/48,844	0.93 (0.75, 1.15)	111/19,180	1.04 (0.80, 1.34)	46/29,664	0.70 (0.47, 1.04)
Alcohol drinking status						
Never drinker	199/84,472	1.00	88/20,730	1.00	111/63,742	1.00
Ever drinker	191/36,314	1.26 (0.98, 1.62)	181/32,145	1.27 (0.97, 1.67)	10/4169	1.23 (0.58, 2.59)
Coffee drinking status						
Never drinker	362/111,234	1.00	248/47,054	1.00	1114/66,921	1.00
Ever drinker	28/9551	0.72 (0.49, 1.06)	21/5821	0.68 (0.43, 1.07)	7/990	0.95 (0.43, 2.10)
Tobacco smoking status						
Never	189/84,642	1.00	74/19,150	1.00	115/65,492	1.00
Ever	201/36,143	**1.48 (1.13, 1.93)**	195/33,725	**1.51 (1.14, 2.01)**	6/2419	1.23 (0.48, 3.17)
Type of tobacco smoking						
Never smoker	189/84,642	1.00	74/19,150	1.00	115/65,492	1.00
Dual smoker	47/9268	1.33 (0.92, 1.93)	46/8681	1.38 (0.94, 2.04)	1/586	0.89 (0.12, 6.76)
Exclusive waterpipe	86/13,539	**1.66 (1.22, 2.28)**	186/2664	**1.75 (1.26, 2.42)**	0/875	-
Exclusive cigarette	68/13,337	1.38 (0.99, 1.91)	163/2380	1.36 (0.96, 1.93)	5/957	2.51 (0.91, 6.86)
History of diabetes						
No	374/118,875	1.00	259/51,954	1.00	115/66,921	1.00
Yes	16/1911	**2.62 (1.58, 4.33)**	10/921	**2.28 (1.20, 4.31)**	6/990	**3.63 (1.59, 8.29)**
Energy (Kcal/day) *	390/151,256	1.01 (0.80, 1.27)	269/83,345	1.16 (0.88, 1.53)	121/67,911	0.73 (0.48, 1.11)
Protein (g/day) *	390/151,256	0.99 (0.82, 1.19)	269/83,345	1.00 (0.80, 1.25)	121/67,911	0.94 (0.67, 1.32)
Fat (g/day) *	390/151,256	0.95 (0.84, 1.08)	269/83,345	**0.86 (0.74, 1.00)**	121/67,911	1.20 (0.95, 1.52)
Carbohydrate (g/day) *	390/151,256	1.04 (0.88, 1.24)	269/83,345	0.94 (0.77, 1.16)	121/67,911	1.34 (0.98, 1.83)
Fiber (g/day) *	390/151,256	1.05 (0.94, 1.17)	269/83,345	1.04 (0.91, 1.19)	121/67,911	1.06 (0.88, 1.27)
Meat (g/week) *	390/151,256	1.01 (0.91, 1.13)	269/83,345	1.06 (0.93, 1.20)	121/67,911	0.92 (0.77, 1.11)
Poultry (g/week) *	390/151,256	1.04 (0.96, 1.13)	269/83,345	1.02 (0.92, 1.13)	121/67,911	1.10 (0.95, 1.27)
Beans (g/week) *	390/151,256	0.99 (0.92, 1.07)	269/83,345	0.99 (0.91, 1.09)	121/67,911	0.98 (0.86, 1.12)
Vegetables (g/week) *	390/151,256	1.08 (0.98, 1.20)	269/83,345	1.06 (0.94, 1.20)	121/67,911	1.13 (0.94, 1.36)
Fruits (g/week) *	390/151,256	0.99 (0.91, 1.09)	269/83,345	0.99 (0.89, 1.11)	121/67,911	1.00 (0.85, 1.17)
Eggs (g/week) *	390/151,256	0.96 (0.90, 1.04)	269/83,345	0.99 (0.90, 1.08)	121/67,911	0.91 (0.79, 1.04)
Fish (g/week) *	390/151,256	0.92 (0.84, 1.01)	269/83,345	0.92 (0.82, 1.02)	121/67,911	0.94 (0.80, 1.10)

Abbreviations: BMI: body mass index; CI: confidence interval; HR: hazard ratio. Multivariable models include indicators (if applicable) of sex, age groups, education, BMI, family history of cancer, alcohol drinking status, coffee drinking status, smoking status, history of diabetes, use of fridge at home, total energy intake, protein intake, fat intake, carbohydrate intake, and fiber intake. * Continuous scale of quintiles. **Bold** font: statistical significance (*p* < 0.05).

**Table 4:** Risk factors for cancer-related deaths in the entire cohort, overall and stratified by sex, based on the Hanoi Prospective Cohort Study.

	Overall		Men		Women	
	# Deaths/ Person-Years	Multivariable Model HR (95% CI)	# Deaths/ Person-Years	Multivariable Model HR (95% CI)	# Deaths/ Person-Years	Multivariable Model HR (95% CI)
Sex						
Men	377/202,445	1.00				
Women	177/224,380	**0.67 (0.53, 0.85)**				
Highest level of education						
Primary school	157/68,035	1.00	85/24,963	1.00	72/43,072	1.00
Secondary school or higher	397/358,789	1.21 (0.97, 1.51)	1292/77,481	1.19 (0.91, 1.55)	105/181,308	1.22 (0.82, 1.80)
Family history of cancer						
No	536/420,521	1.00	366//199,523	1.00	170/220,998	1.00
Yes	18/6304	**4.57 (2.82, 7.43)**	11/2922	**5.04 (2.73, 9.30)**	7/3382	**4.68 (2.05, 10.68)**
BMI						
18.5–22.9	299/225,337	1.00	206/108,032	1.00	93/117,305	1.00
<18.5	46/34,083	0.76 (0.55, 1.03)	28/20,379	0.62 (0.42, 0.92)	18/13,704	1.31 (0.79, 2.17)
≥23	209/167,404	0.87 (0.72, 1.04)	143/74,034	0.90 (0.73, 1.12)	66/93,370	0.69 (0.50, 0.96)
Alcohol drinking status						
Never drinker	279/328,980	1.00	112/112,833	1.00	167/216,147	1.00
Ever drinker	275/97,844	**1.55 (1.25, 1.92)**	265/89,611	**1.68 (1.33, 2.14)**	10/8233	1.03 (0.50, 2.14)
Coffee drinking status						
Never drinker	517/391,761	1.00	348/181,156	1.00	169/210,605	1.00
Ever drinker	37/35,064	**0.64 (0.45, 0.90)**	2921,289	**0.67 (0.46, 0.99)**	8/13,775	0.55 (0.25, 1.19)
Tobacco smoking status						
Never	263/327,409	1.00	92/107,433	1.00	171/219,976	1.00
Ever	291/99,415	**1.87 (1.48, 2.36)**	285/95,012	**2.01 (1.56, 2.59)**	6/4404	1.10 (0.43, 2.79)
Type of tobacco smoking						
Never smoker	263/327,409	1.00	92/107,433	1.00	171/219,976	1.00
Dual smoker	74/28,912	**1.72 (1.26, 2.34)**	72/27,794	**1.86 (1.35, 2.58)**	1/1118	-
Exclusive waterpipe	126/32,473	**2.27 (1.74, 2.97)**	126/30,924	**2.49 (1.87, 3.32)**	0/1549	-
Exclusive cigarette	91/38,031	**1.60 (1.20, 2.12)**	86/36,294	**1.67 (1.23, 2.27)**	5/1737	2.27 (0.84, 6.14)
History of diabetes						
No	537/424,505	1.00	366/201,304	1.00	171/223,201	1.00
Yes	17/2320	**2.62 (1.61, 4.27)**	11/1141	**2.39 (1.30, 4.38)**	6/1178	**3.20 (1.41, 7.28)**
Energy (Kcal/day) *	554/457,293	0.98 (0.81, 1.18)	377/232,914	1.13 (0.90, 1.42)	177/224,379	0.72 (0.51, 1.01)
Protein (g/day) *	554/457,293	1.00 (0.86, 1.16)	377/232,914	0.96 (0.80, 1.15)	177/224,379	1.09 (0.83, 1.44)
Fat (g/day) *	554/457,293	0.89 (0.80, 0.99)	377/232,914	**0.85 (0.75, 0.97)**	177/224,379	0.99 (0.82, 1.20)
Carbohydrate (g/day) *	554/457,293	1.11 (0.96, 1.28)	377/232,914	1.04 (0.87, 1.23)	177/224,379	1.26 (0.98, 1.63)
Fiber (g/day) *	554/457,293	1.03 (0.95, 1.13)	377/232,914	1.02 (0.91, 1.13)	177/224,379	1.06 (0.91, 1.24)
Meat (g/week) *	554/457,293	1.00 (0.91, 1.09)	377/232,914	1.03 (0.93, 1.14)	177/224,379	0.93 (0.80, 1.09)
Poultry (g/week) *	554/457,293	1.01 (0.94, 1.08)	377/232,914	0.98 (0.90, 1.06)	177/224,379	1.08 (0.97, 1.22)
Beans (g/week) *	554/457,293	1.01 (0.95, 1.07)	377/232,914	1.01 (0.93, 1.08)	177/224,379	1.00 (0.90, 1.12)
Vegetables (g/week) *	554/457,293	1.07 (0.99, 1.17)	377/232,914	1.07 (0.97, 1.19)	177/224,379	1.07 (0.92, 1.24)
Fruits (g/week) *	554/457,293	0.99 (0.92, 1.07)	377/232,914	1.01 (0.92, 1.10)	177/224,379	0.96 (0.84, 1.10)
Eggs (g/week) *	554/457,293	1.00 (0.94, 1.06)	377/232,914	1.02 (0.94, 1.10)	177/224,379	0.96 (0.86, 1.07)
Fish (g/week) *	554/457,293	0.95 (0.88, 1.02)	377/232,914	0.95 (0.87, 1.04)	177/224,379	0.95 (0.83, 1.09)

Abbreviations: BMI: body mass index; CI: confidence interval; HR: hazard ratio. Multivariable models include indicators (if applicable) of sex, age groups, education, BMI, family history of cancer, alcohol drinking status, coffee drinking status, smoking status, history of diabetes, use of fridge at home, total energy intake, protein intake, fat intake, carbohydrate intake, fiber intake. * Continuous scale of quintiles. **Bold** font: statistical significance (*p* < 0.05).

## Data Availability

The data presented in this study are available on request from the corresponding author due to ethical reasons.

## Supplementary Materials

The following supporting information can be downloaded at https://www.mdpi.com/article/10.3390/cancers16223853/s1: Supplemental methods; Figure S1: Eligible study participants aged less than 50 years and 50 years or older; Figure S2: Kaplan–Meier survival estimates by alcohol drinking status for young adults aged under 50 (A) and those aged 50 years or older (B) and by smoking status (C) and (D); Table S1: population attributable risk fraction of deaths due to dual smoking, exclusive waterpipe smoking, exclusive cigarette smoking, alcohol drinking, and ever smoking in young adults and persons aged 50 years or older.
