# Supplementary material for: Factors Affecting Cancer Mortality in Young Adults: Findings from a Prospective Cohort Study
Source: Cancers (Basel). 2024 Nov 17;16(22):3853. doi: 10.3390/cancers16223853 (PMC11593055; doi:10.3390/cancers16223853)
Supplement: Supplementary file 1 [file cancers-16-03853-s001.zip › cancers-3282637-supplementary.pdf]

cancers MDPI

Supplemental Online Content

Ngoan Tran Le, Yen Thi-Hai Pham, Linh Thuy Le, Hang Viet Dao, Chihaya Koriyama, Toan H. Ha, Maureen Lichtveld, Suresh Kuchipudi, Nhi Yen Ngoc Huynh, Dai Duc Nguyen, Hung N. Luu

Supplemental Methods

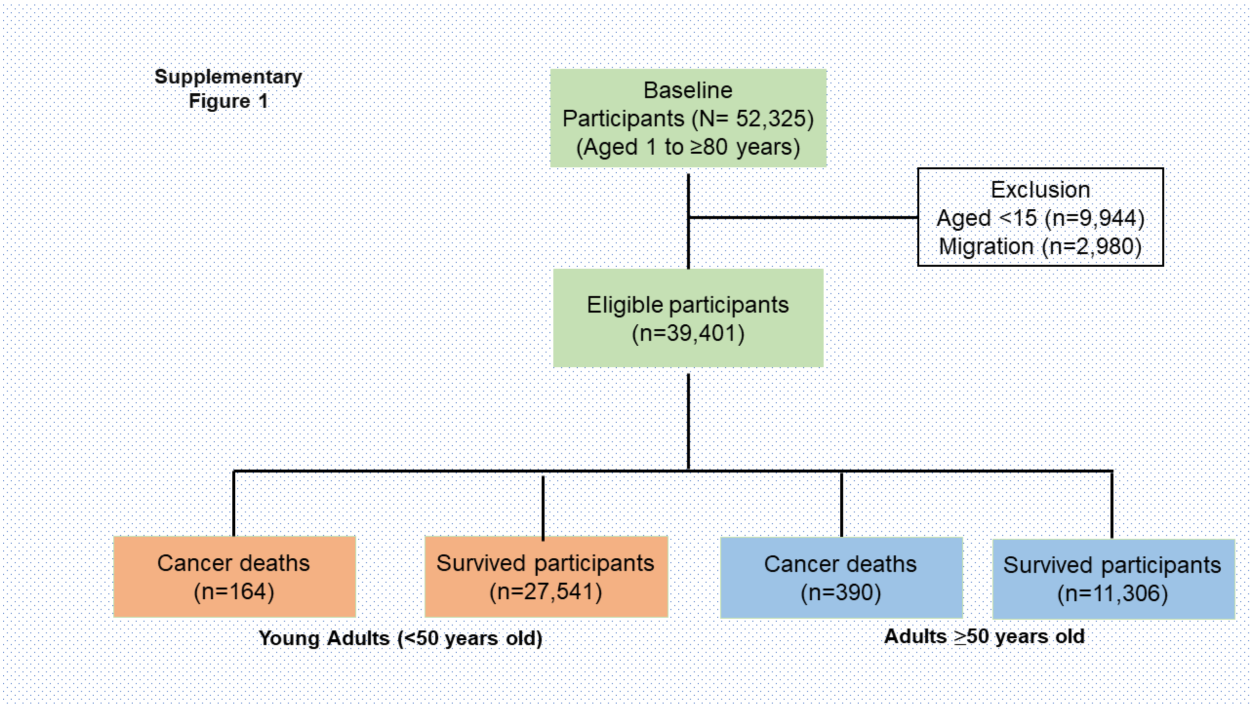

Figure S1. Eligible study participants aged less than 50 years and 50 years or older.

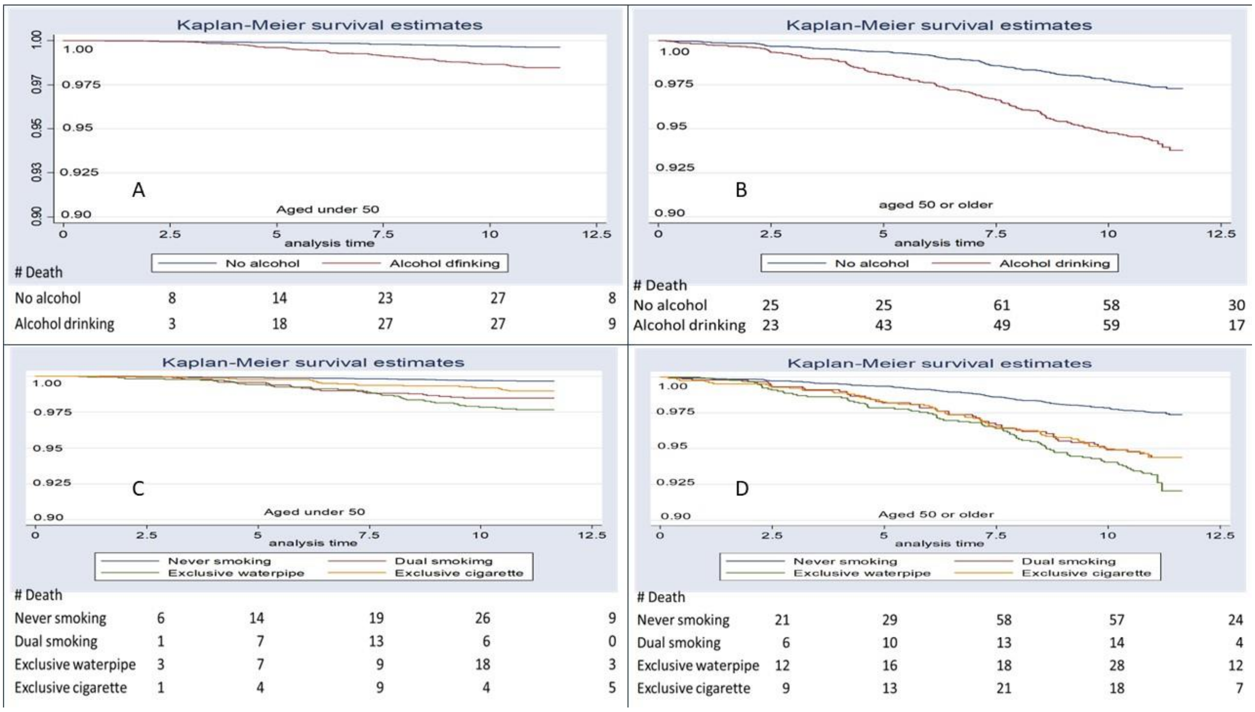

Figure S2. Kaplan-Meier survival estimates by alcohol drinking status for young adults aged under 50 (A) and those aged 50 years and older (B), and by smoking status (C) and (D), respectively. This supplemental material has been provided by the authors to give readers additional information about their work.

Table S1. Population Attributable Risk Fraction of Deaths due to Dual Smoking, Exclusive Waterpipe Smoking, Exclusive Cigarette Smoking, Alcohol Drinking and Ever Smoking in Young Adults and Person Aged 50 Years or Older.

**Factors**	**Young Adults**			**Adults ≥50 Years Old**			**Combined PAF (%)**
	**Prevalence (%)**	**HR**	**PAF (%)**	**Prevalence (%)**	**HR**	**PAF (%)**	
**Dual smoking**	12.97	3.13	21.65	16.43	1.38	5.88	15.77
Exclusive waterpipe smoking	12.31	4.28	28.76	24.10	1.75	15.31	13.46
Exclusive cigarette smoking	15.93	2.39	18.13	22.93	1.36	7.63	10.50
Alcohol drinking	38.68	2.67	39.25	60.61	1.27	14.06	25.18
Ever smoking	41.2	2.55	38.97	63.45	1.51	24.45	14.52

Supplemental Methods

Smoking Assessment

The structured questionnaire, aka. a designed demographic lifestyle and semi-quantitative food frequency questionnaire (DLQ and FFQ), was used to collect information on tobacco smoking, diet, and related information. Two main types of tobacco, including cigarette and waterpipe, were collected in the Hanoi Prospective Cohort Study.[34] To collect detailed information on cigarette smoking, we used the

following questions: 1) *"Do you currently smoke cigarettes every day?"* (Yes/no); if "yes," *"What is the average number of cigarettes smoked daily?" "Number of years smoked?"*; 2) *"Do you currently smoke cigarettes weekly?"* (Yes/no); if *"yes," "what is the average number of cigarettes smoked weekly?" "Number of years smoked?"*; 3) *"If you do not currently smoke cigarettes daily or weekly, in the past, did you smoke cigarettes daily?"* (Yes/no); if *"yes," "what is the average number of cigarettes smoked daily?" "Number of years smoked?" "The number of years you have quit smoking?"*. Similar types of questions were used to obtain detailed information on waterpipe smoking. A validation study on a smoking questionnaire among 147 participants was conducted twice; each interview was two weeks apart to determine the agreement between those interviews. Two independent and experienced interviewers performed the interviews. The respective kappa statistics (κ) and 95% confidence interval (CI) values, representing the agreement between two independent results of current and former tobacco smoking of all tobacco types (i.e., cigarette and waterpipe), were as high as 96.58% (93.64-99.52), $P<0.01$) and 95.80% (92.56 - 99.04), $P<0.01$.

The current analysis categorized smoking into 1) ever smoker and 2) never smoker. Never-smoker was defined as those who had never smoked tobacco of any type. Ever smoker was defined as those who smoked any tobacco, including cigarettes, waterpipe, or dual smokers. In addition, we also defined former smokers as those who have smoked in their lifetime but did not smoke for six months or longer before the interview date. Smoking frequency was smoking intensity daily reported during any age period. Cumulative smoking was calculated by multiplying the average daily smoking session and the duration of tobacco, represented by years. Smoking session (years) among past smokers was calculated from the year they indicated that they stopped smoking successfully to the interview date. We compared the proportions of every tobacco smoker between the current dataset (after the exclusion of 2,980 lost to follow-up

participants) and the dataset before such exclusion to see if there was a significant discrepancy. The difference between these two proportions was 0.44% (P-*value*=0.09). This suggests that the exclusion of 2,980 lost to follow-up participants did not materially change the smoking status of our current analysis.

Cancer Deaths Ascertainment

All-cause of mortality information, including cancer-related deaths, was identified based on medical records available at the CHS, district hospitals, provincial hospitals, and other health facilities. Detailed information regarding mortality included deceased date, month and year, place of final medical examination and diagnosis, treatment, and medical certificate issued by the health facility. We used the International Classification of Diseases, Tenth Revision (ICD-10) code for cause-specific mortality in our cohort, including cancer deaths. Palliative care for cancer patients living in Hanoi City before the end event was admitted into Central Hospitals (64.3%), City Hospitals (24.1%), Private Hospitals (1.3%), and no available data on palliative care (5.1%).[50] The staff of each CHS (or Family Doctors) transferred cancer patients to the higher facility hospitals and continued palliative care at the local CHS until the end of health events. From this service, causes of death from cancer were identified and checked. For the initial mortality registry, validation of mortality data showed that completeness, sensitivity, and specificity were 93.9%, 75.4%, and 98.4%, respectively. An advanced search, consultations, and independent second opinion of the underlying cause of death was performed to clarify the cause of cancer death for each case in the list of false negatives (24.6%) and incompleteness (6.1%). In the present study, 554 cancer deaths were identified. For those who migrated out, we collected the following information: 1) the move date (day, month, and year) and 2) the new address.

In the current analysis, the last follow-up was on December 31, 2019, or at the time when the information on those who died or had events or moved out of the community was confirmed. Follow-up time was defined by years from enrolment to the date of death, loss-to-follow-up, or end of follow-up, whichever came first.
